# Hand-in-hand in the golden years: Cognitive interdependence, partner involvement in retirement planning, and the transition into retirement

**DOI:** 10.1371/journal.pone.0261251

**Published:** 2021-12-29

**Authors:** Veronica M. Lamarche, Jonathan J. Rolison

**Affiliations:** Department of Psychology, University of Essex, Colchester, Essex, United Kingdom; Flinders University, AUSTRALIA

## Abstract

This research examined the influence of cognitive interdependence—a mental state reflecting a collective representation of the self-in-relationship—on the anticipation for and experiences with the transition into retirement. Among soon-to-be retirees (Study 1), greater cognitive interdependence was associated with seeing partners as more instrumental to one’s goals both pre- and post-retirement, anticipating greater goal alignment post-retirement, and having directly involved partners in retirement planning to a greater extent than those relatively lower in cognitive interdependence. Among recent retirees (Study 2), retrospective cognitive interdependence was associated with post-retirement goal alignment and goal instrumentality, and the extent to which they believed they had directly involved their partners in retirement planning. However, it was post-retirement goal alignment that was associated with greater ease of retirement and subjective well-being. Finally, soon-to-be retirees relatively high in cognitive interdependence responded to concerns about their retirement (i.e., goal discordance and high retirement ambivalence) by wanting to involve their partners in their retirement plans to a greater extent (Study 3). These studies highlight the importance of romantic partners across the lifespan, and how partners might influence retirement planning, the transition to retirement, and well-being among recent retirees.

## Introduction

Many developed nations are experiencing new types of growing pains as an unprecedented number of older adults in these “graying societies” begin their transitions into retirement (e.g., [1]). These sweeping sociodemographic changes have led to renewed interest into the factors that contribute to well-being and happiness following the transition into retirement. Although many people look forward to their retirement, like other major life events, the transition to retirement can be both transformative and disruptive. People may be financially prepared for life post-retirement, but may nonetheless be underprepared for the stress associated with changing social roles and identities. During this period, romantic partners can either be a salve or a source of further stress depending on whether they make the transition to retirement easier or more challenging (e.g., [2, 3]). People differ in the extent to which they integrate their relationship into their sense of self [4]. The integration of self-in-relationship helps people to believe that they share the same reality [5], allows couples to operate as a single self-regulating unit [6], and behave more prosocially and communally for the benefit of the couple over the individual [7]. This collective self-identity should therefore influence the extent to which people involve their partners in their retirement planning decisions, with important consequences for after they retire [8]. The current studies examine whether people who experience greater cognitive interdependence (i.e., the extent to which people have a mental representation of their self-identity as collectively and pluralistically integrated into their romantic relationship) anticipate and experience easier transitions into retirement than those in less interdependent partnerships, and whether cognitive interdependence is associated with a person more directly involving their partner in their retirement planning decisions.

### The challenge of the transition into retirement

Successfully planning for retirement can be a daunting and overwhelming challenge. Not only do people need to correctly anticipate what their financial needs will look like long after they stop working, but they must also anticipate and prepare for changes to their identity and social networks [9, 10]. Many people experience acute declines in well-being and life satisfaction after retirement [11]. These declines are associated with lost meaning and purpose in life [12], a disrupted sense of self [13], and increased interpersonal problems [14], all of which are associated with poorer physical and psychological health [11]. Although retirement planning advice exists, this guidance focuses almost exclusively on how to achieve financial security in the future [15]. However, for many adults, retirement is not a solitary endeavor, as most people plan on sharing their golden years with a romantic partner. People rely heavily on their romantic partners to successfully navigate major life events and transitions, including marriage, first-time parenthood, career changes, and coping with acute and chronic illnesses [16]. Thus, during the transition into retirement, partners not only influence when people decide to retire [17], but they should also play a crucial role in shaping what people’s goals for retirement look like and facilitating these goals along the way [18]. In order to optimize positive outcomes in retirement, it is both necessary to begin planning effectively well in advance and to draw upon and strengthen sources of social support. However, the extent to which people are likely to include their partner in retirement planning should depend on whether or not they perceive their partner as someone who will facilitate their goals or impede them.

### From me to we: Cognitive interdependence

As relationships persist, people become increasingly enmeshed and integrated into each other’s lives, and exert a mutually strong influence on each other [19, 20]. This mutual influence is known as interdependence and informs how people think and react across interpersonal situations with or without the presence of the partner [21]. Cognitive interdependence reflects the extent to which the relationship is represented mentally as a collective and pluralistic representation of the self-in-relationship [7]. These pluralistic self-concepts lead people to view their partners as more central and instrumental in their lives as the relationship becomes more integral to their self-identity [22, 23]. Greater interdependence motivates people to behave more communally and prioritize behaviors which benefit the relationship over the self [24, 25], and is associated with relationship-enhancing behaviors including more effective communication strategies, greater willingness to accommodate and forgive, and more reciprocity towards the partner [26–28]. This relationship-focused perspective has important consequences for the individuals within these relationship structures. For instance, interdependence has been associated with people making more communally-motivated risk-reducing health decisions which benefit the relationship, instead of prioritizing their own self-interest [29]. Similarly, communal coping strategies associated with interdependence have been linked with more diabetes problem-solving, positive mood and greater relationship quality among couples where one partner has recently been diagnosed with type 2 diabetes [30]. Interdependence is therefore a valuable resource in times where couples must navigate potentially conflicting needs and desires (e.g., different priorities for retirement), because it motivates people to prioritize communally-focused and relationship-centered behaviors. Thus, even in periods of uncertainty, cognitively interdependent people should be more willing to involve their partners in their retirement plans than those in less interdependent partnerships.

### Working hand-in-hand: Goal pursuit and relationships

Living an interconnected life means that people’s goals, actions, and behaviors exist in close connection with those of their romantic partner [19]. Partners help each other coordinate goals and facilitate achieving goals [6], strive toward growth and self-improvement [31], and provide one another’s lives with meaning and purpose [32, 33]. Reliable access to partner support is therefore critical for well-being and successful goal pursuit [34]. In fact, the positive effect goal facilitation by close others has on personal well-being is suggested by some to be the reason why relationships are such a valuable personal resource [35]. This is especially salient during the transition to retirement when people experience sudden changes to their, often shrinking, social networks [10].

Partners are said to be instrumental to goals insomuch as they actively facilitate goal pursuits and increase the likelihood of people attaining their goals [36, 37]. This instrumentality leads couples to function as a single goal-pursuing unit, even when working toward personal goals, and contributes to feelings of goal alignment (i.e., complementary rather than conflicting goals; [6]). However, goals are not always aligned, which means that during the transition to retirement, romantic partners may not necessarily share the same vision for their lives following retirement. For example, a 2015 survey of over 1,000 couples found that nearly half respondents disagreed with their partner’s assessment of how much they needed to save for retirement, despite also reporting that they were well-prepared for retirement [38]. This discrepancy between feeling prepared despite having misaligned expectations and goals highlights the reality that many couples are not coordinated in their retirement plans. This discordance is problematic because the extent to which partners can help one another set clear retirement goals, and effectively pursue them, is associated with better outcomes such as greater savings contributions, well-being, and relationship quality [8, 39, 40]. Thus, regardless of whether the plans are financial (e.g., savings), social (e.g., spending more time traveling as a couple vs. more time with the grandkids), or self-actualizing (e.g., starting a new hobby), people with partners who can more successfully navigate these interdependence dilemmas and coordinate their retirements with their partners should be in a better position to avoid conflict and plan more effectively for their retirements than those who withdraw from their partner at the first sign of stress or disagreement.

### Interdependence, goals and successful transitions into retirement

The transition into retirement should therefore be influenced by the extent to which feeling cognitively interdependent with the partner increases the likelihood that people believe they have goals for the future which are aligned with their partners’, as well as the extent to which people involve their partners in planning decisions. As partners facilitate one another’s goals, their own goals become interconnected; the dyad begins to act as a single self-regulating system, pursuing self-, partner- and relationship-oriented goals simultaneously [6]. When people feel interconnected with their partners, they conflate the boundaries between “self” and “partner” [4], and assume partners operate within a shared reality, which both stems from engaging in joint activities as well as continuing to motivate partner involvement [5]. Furthermore, greater goal alignment is associated with an increased likelihood of engaging the partner in joint planning decisions, which is associated with a lower likelihood of divorce [8] and enhanced general well-being [40]. In the context of retirement planning, high levels of cognitive interdependence should lead people to believe that their partners share aligned goals following retirement. This should motivate people to solicit their partner’s advice and directly involve them in retirement planning decisions, which will in turn help ensure greater actual goal alignment in the future. Thus, greater cognitive interdependence should facilitate the transition into retirement: more collective representations of the self-and-partner (i.e., cognitive interdependence) should motivate people to believe partners have shared goals and futures, which should in turn elicit behaviors (e.g., direct planning) that facilitate shared outcomes (i.e., goal alignment and goal instrumentality in retirement). Seeing the self-and-relationship as more interconnected should also help people to respond more collectively and pro-relationally to the inevitable conflicts that arise as people coordinate their long-term goals. Furthermore, greater coordination and shared realities in retirement should be associated with greater ease of retirement and higher subjective well-being.

### Current research

Effective planning for retirement is paramount for successfully transitioning into retirement. However, because goals are typically pursued in the presence of others, the extent to which partners are seen as more collectively interconnected with personal outcomes should have important consequences for how people plan for their retirements as well as how they experience them. Thus, in the current research, we investigated whether cognitive interdependence was positively associated with pre-retirement expectations and post-retirement experiences, as a consequence of greater retirement goal alignment and having directly involved their partners in their retirement planning decisions. Further, we explored whether these factors were associated with greater personal well-being in retirement. The current research also tested whether cognitive interdependence influenced how people coped with uncertainty surrounding their future retirement.

We tested these associations in two correlational studies—one with older adults approaching retirement (Study 1) and one with recent retirees (Study 2)—and one experimental paradigm (Study 3). In the absence of a single longitudinal study tracking couples across the transition into retirement, the correlational studies provide important insights into the thoughts and behaviors of people approaching and recently entering retirement. Successfully planning for retirement requires that people make optimal decisions *before* they retire, particularly with regards to aligning goals and expectations with another person (i.e., the partner). Thus, Study 1 provides insight into the interpersonal characteristics that are associated with engaging the partner in planning decisions prior to having retired. Study 2 provides insight into whether these same interpersonal characteristics and behaviors remain important after the transition to retirement has occurred.

In Study 1, we expected that greater cognitive interdependence would be associated with greater perceived partner goal instrumentality, more direct involvement of the partner in retirement planning decisions, greater anticipated goal alignment in retirement, and ultimately with an easier anticipated transition into retirement. In Study 2, we tested whether greater retrospective cognitive interdependence was associated with having directly involved partners in retirement planning to a greater extent, and post-retirement goal alignment and instrumentality, and ultimately if it was associated with more positive retirement experiences and higher well-being among recent retirees. We expected that the same pattern of findings hypothesized for Study 1 to replicate in Study 2 post-retirement, specifically that higher retrospective cognitive interdependence would be associated with higher reported partner involvement in retirement planning decisions, higher reported goal alignment post-retirement, higher reported ease of the transition into retirement, and higher subjective well-being. Finally, in Study 3, we experimentally tested whether people with greater cognitive interdependence in their relationship coped with uncertainty about their retirements (e.g., goal conflicts with the partner; ambivalence about retirement) using communally-centered responses by involving their partners in their retirement planning to a greater extent. We expected a three-way interaction between cognitive interdependence, goal conflicts and retirement ambivalence predicting higher partner involvement in retirement planning. We expected that participants who reported higher cognitive independence would report higher partner involvement in their future retirement plans in the presence of goal conflict and retirement ambivalence, compared to participants who reported lower cognitive interdependence. The survey materials, aggregate data, and syntax for reproducing the analyses for these studies are available online via the project’s Open Science Framework page (https://osf.io/jgh35).

## Study 1

Study 1 examined whether greater cognitive interdependence was associated with (a) greater pre- and post-retirement goal instrumentality; (b) greater perceived alignment of goals post-retirement; (c) having more directly involved their partner in their retirement planning; and, (d) more positive expectations about the ease of their transition into retirement and changes to their relationship.

### Methods

#### Participants

There is not a mandatory retirement age in the United States (US), however the mean retirement age is 61 years old, and people do not qualify for Medicare (public health coverage) until 65 years old and full social security until 67 years old. We were interested in targeting participants who were likely to retire soon and should therefore be more actively engaged in retirement planning (compared to relatively younger people for whom retirement is a distant life event), and therefore restricted recruitment to adults between the ages of 50 and 70. One hundred and four employed adults aged 52–70 years based in the US, and currently in a romantic relationship, were recruited via Amazon’s Mechanical Turk (MTURK) to take part in this study online (prescreening questions can be found in the survey materials on the project’s OSF page). Although recruitment was open to participants between the ages of 50 and 70, none of the eligible participants who took part in our study were between the ages of 50 and 51. The quality and reliability of MTURK data is comparable with other testing methods (e.g., face-to-face behavioral testing) and the MTURK platform provides a diverse sample in terms of socioeconomic characteristics [41]. Twelve participants were excluded for being under the age of 50 (n = 4) or over the age of 70 (n = 5) when asked to confirm their year of birth following pre-screening, for identifying as single/not in a romantic relationship (n = 1), and for not completing the survey to completion (n = 2), leaving a final sample of 92 participants (*M*_*age*_ = 58.85; *SD* = 5.00; 53% men) upon which all reported analyses are based. Participants were within 10-years to their planned retirements on average (*M*_*years*_ = 8.71; *SD =* 6.27). Participants had been in relationships an average of 20.93 years (*SD =* 14.59), and the majority (72%) were married (21% exclusively dating/in a committed relationship; 4% casually dating; 2% engaged). Participants came from different educational backgrounds (14% high school graduate; 15% some college but no degree; 15% associate’s degree (2-year); 36% bachelor’s degree (4-year); 16% master’s degree; 2% doctoral degree; 1% professional degree), and employment sectors (18% sales, distribution & retail; 16% information technology, legal or management services; 19% other services industry; 10% education; 10% healthcare and medical; 9% finance and banking; 6% manufacturing; 3% civil services and local government; 3% primary industries [farming; fishing; mining, etc.]). The majority (39%) of our participants earned between $50,000 and $99,999 per year, with the rest of the participants earning less than $29,999 per year (16%), between $30,000 and $49,999 per year (19%), between $100,000 and $149,999 per year (17%), or over $150,000 (7%).

#### Materials & procedures

Following the demographics questionnaire, participants completed the following measures. This research received ethical approval from the University of Essex Ethics Subcommittee 3. Participants gave written consent to take part in the study; they were provided with an information sheet and consent form on the first page of the survey, before the screening questions. Participants who did not select “I consent” from the list on the consent form immediately had their session terminated and were thanked for their time and interest.

*Cognitive interdependence*. Cognitive interdependence with the partner was assessed using a 4-item measure [7] (*α* = .87; “In comparison to other parts of your life [e.g., work, family, friends, religion], how central is your relationship with your partner”) rated on a 7-point scale (1 = not at all central, 7 = extremely central). Higher mean scores reflect greater cognitive interdependence.

*Goal instrumentality*. Participants rated how much their partner currently helps them pursue goals across life domains (i.e., social, romantic, financial, career, health/fitness, leisure/fun, personal improvement/growth, service/helping others) pre-retirement and post-retirement (adapted from [37]). Ratings were made on an 11-point scale (-5 = extremely harmful, 0 = neither helpful nor harmful, 5 = extremely helpful) with high scores reflecting greater goal instrumentality (pre-retirement, *α* = .92; post-retirement, *α* = .94).

*Goal alignment*. A single-item measure assessed perceived post-retirement goal alignment (“How well aligned do you feel your post-retirement goals align with your partner’s post-retirement goals”; 1 = completely unaligned/we have different goals, 7 = completely aligned/we have the same goals).

*Partner involvement in retirement planning*. A single-item measure assessed how much participants had directly involved their partners in planning decisions for after they retired. Ratings were made on a 7-point scale (1 = my partner has not been directly involved in planning at all, 7 = my partner has been directly involved in planning a great deal), with higher mean scores indicating greater partner involvement.

*Anticipated ease of retirement*. A single-item measure assessed how easy participants expected their transition into retirement to be (“How easy do you expect your transition to retirement to be?”; 1 = extremely difficult, 7 = extremely easy).

*Self-esteem*. Higher self-esteem is associated with more positive views of partners and relationships, greater optimism, and greater perceived self-efficacy and confidence. Thus, in order to isolate the influence of cognitive interdependence on retirement outcomes, self-esteem was included as a covariate in the analyses to control for the possibility of a general positivity bias in the effects. A 10-item measure [42] assessed trait self-esteem (e.g., “I feel that I am a person of worth, at least on an equal basis with others). Ratings were made on a 7-point scale (*α* = .93; 1 = strongly disagree, 7 = strongly agree), with higher mean scores reflecting higher self-esteem.

### Results

#### Descriptives

On average, participants reported that they were highly interdependent (*M =* 5.72, *SD =* 1.02), that their partners were generally instrumental to their goals across domains (*M* = 1.93, *SD* = 1.86), and that they believed their post-retirement goals were relatively aligned with their partner’s (*M* = 5.60, *SD* = 1.32). Likewise, participants on average reported that they had largely directly involved their partners in their retirement plans thus far (*M* = 5.43, *SD* = 1.42). Finally, participants anticipated a relatively easy transition into retirement (*M* = 5.23, *SD* = 1.64). Table 1 presents the descriptive statistics for the measures in this study and their correlations.

**Table 1:** Study 1 correlations and descriptive statistics.

Measure	1	2	3	4	5	6	7	8	9	10	11
1. Cognitive Interdependence	--										
2. Pre-Retirement Goal Instrumentality	.61***	--									
3. Post-Retirement Goal Instrumentality	.69***	.91***	--								
4. Goal Alignment	.40***	.69***	.66***	--							
5. Partner Involvement	.40***	.57***	.55***	.53***	--						
6. Ease of Retirement	.41***	.48***	.45***	.48***	.49***	--					
7. Relationship Change	.49***	.57***	.60***	.38***	.33**	.38***	--				
8. Self-Esteem	.33**	.49***	.44***	.38***	.37***	.22*	.13	--			
9. Gender	-.05	.06	.02	.03	.12	-.06	-.25*	.21*	--		
10. Years to Retirement	-.14	-.25*	-.18†	-.32**	-.25*	-.40***	-.17†	-.27**	.06	--	
11. Age	.28**	.30**	.26*	.34**	.25*	.16	.16	.28**	.03	-.55***	--
*M*	5.72	1.93	2.00	5.42	5.23	4.82	1.02	5.60	--	8.71	58.85
*SD*	1.02	1.86	2.19	1.42	1.64	1.54	1.38	1.32	--	6.27	5.00
*Observed Range*	1.50–7	-3.86–5	-4.71–5	1–7	1–7	1–7	-3-3	1.90–7	--	0–25	52–70

Note.

†p<0.1

*p < .05

**p < .01

*** p < .001. Table 1 presents the bivariate correlations between measures, as well as their mean (M), standard deviation (SD), and observed range in the sample.

#### Primary analyses

We hypothesized that soon-to-be retirees who were more cognitively interdependent in their relationship would see their partners as more instrumental and aligned with their retirement goals, and would be more likely involve their partners directly in their retirement planning decisions. We also hypothesized that people who were more cognitively interdependent would anticipate an easier transition into retirement. Regression analyses were used to predict these outcomes from cognitive interdependence, controlling for gender, years until retirement, and individual differences in self-esteem as covariates (see Table 2 for model coefficients). R (v. 4.0.5) was used for all analyses in this study.

**Table 2:** Model coefficients: Study 1.

	Predictor		Covariates					
	Cognitive Interdependence		Self-Esteem		Gender		Years to Retirement	
**Dependent Variables**	***b***	***t***	***b***	***t***	***b***	***t***	***b***	***t***
Partner Involvement in Planning	.*51*	*3*.19**	.*25*	*1*.*92*†	.*33*	*1*.*07*	*-*.*04*	*-1*.*61*
Pre-Retirement Goal Instrumentality	.*91*	*5*.95***	.*41*	*3*.31**	.*08*	.*26*	*-*.*03*	*-1*.*26*
Post-Retirement Goal Instrumentality	*1*.*31*	*7*.67***	.*38*	*2*.72**	*-*.*02*	.*97*	*-*.*01*	.*60*
Anticipated Post-Retirement Goal Alignment	.42	3.07**	.24	2.17*	.02	.06	-.05	-.05
Anticipated Ease of Retirement	.55	3.72***	.01	.11	-.08	-.27	-.08	-3.69***

Note.

†p<0.1

*p < .05

**p < .01

*** p < .001.

Table 2 presents the regression model coefficients for cognitive interdependence predicting partner involvement in retirement planning, pre-retirement goal instrumentality, post-retirement goal instrumentality, anticipated post-retirement goal alignment, and anticipated ease of retirement, controlling for trait self-esteem, gender and years to retirement as covariates.

As hypothesized, cognitive interdependence was significantly and positively associated with pre- and post-retirement goal instrumentality, anticipated post-retirement goal alignment, having directly involved the partner in retirement planning decisions, and an easier anticipated transition to retirement (*ps <* .*01*). Although a traditional alpha criterion of *p* < .05 is used to interpret the findings, these hypothesis tests also pass the Bonferroni corrected alpha criterion threshold for significance of *p* < .01 for this study.

## Study 2

The goal of Study 2 was to examine whether the associations found in Study 1, regarding cognitive interdependence, goal instrumentality and alignment, and partner involvement in retirement planning persist among people who have recently retired, and whether they were associated with greater well-being in retirement.

### Methods

#### Participants

Ninety-seven adults over the age of 60 based in the United States who were currently in a romantic relationship and who had retired were recruited via Amazon MTURK to take part in this study online (prescreening questions can be found in the survey materials on the project’s OSF page). Nine participants were excluded for reporting they were not in a relationship (n = 2), for being under 60 years old (n = 3) when asked to confirm their year of birth following pre-screening, for not identifying as a man or woman (n = 1) and for not completing the survey through to the end (n = 3) leaving a final sample of 88 participants (*M*_*age*_ = 68.08, *SD* = 3.90; 53% women). Participants had been in their relationships for an average of 33.12 years (*SD* = 18.57) and the majority (82%) were married (3% casually dating, 15% in an exclusive/committed dating relationship). Participants came from different education backgrounds (11% high school graduate; 23% some college but no degree; 13% associate’s degree (2-year); 31% bachelor’s degree (4-year); 16% master’s degree; 0% doctoral degree; 4% professional degree), and employment sectors (13% sales, distribution & retail; 9% information technology, legal or management services; 18% other services industry; 13% education; 13% healthcare and medical; 10% finance and banking; 9% manufacturing; 6% civil services and local government; 2% primary industries [farming; fishing; mining, etc.]; 1% armed forces). The majority (56%) of our participants earned between $50,000 and $99,999 per year, with the rest of the participants earning less than $29,999 per year (6%), between $30,000 and $49,999 per year (30%), between $100,000 and $149,999 per year (7%), or over $150,000 (1%).

#### Materials & procedure

This research received ethical approval from the University of Essex Ethics Subcommittee 3. Participants gave written consent to take part in the study; they were provided with an information sheet and consent form on the first page of the survey, before the screening questions. Participants who did not select “I consent” from the list on the consent form immediately had their session terminated and were thanked for their time and interest. Participants completed a retrospective measure of cognitive interdependence from Study 1 and reflected on the changes in interdependence since retirement. Next, participants were asked to reflect on how instrumental their partner was to their goals post-retirement, how well aligned they felt their current post-retirement goals are with their partner’s post-retirement goals, how actively they involved their partners in retirement planning prior to retirement, how easy their transition into retirement had been, and self-esteem, using the same measures from Study 1. Additionally, participants in Study 2 completed a measure of current well-being.

*Cognitive interdependence*. Retrospective cognitive interdependence with the partner was assessed using a 4-item measure [7] (*α* = .89; “In comparison to other parts of your life [e.g., work, family, friends, religion], how central is your relationship with your partner **before** you retired”) rated on a 7-point scale (1 = not at all central, 7 = extremely central). Higher mean scores reflect greater (retrospective) cognitive interdependence.

*Changes in cognitive interdependence*. Participants also completed a modified version of the 4-items from the cognitive interdependence scale which assessed the extent to which they felt their interdependence had become stronger or weaker since retirement (*α* = .90; “Thinking about your relationship with your partner before you retired compared to since you have retired: How central is your relationship with your partner now compared to before you retired”), rated on a 7-point scale (1 = a lot less central now, 7 = a lot more central now). Higher mean scores reflect greater perceived cognitive interdependence now relative to before retirement.

*Post-retirement goal instrumentality*. Participants completed the same 11-item measure of goal instrumentality post-retirement as Study 1 (*α* = .91; -5 = extremely harmful, 0 = neither helpful nor harmful, 5 = extremely helpful).

*Post-retirement goal alignment*. Participants completed the same single-item measure of goal alignment as Study 1, rephrased to reflect current goal alignment (“How well aligned do you feel your post-retirement goals align with your partner’s retirement goals”; 1 = completely unaligned/we have different goals, 7 = completely aligned/we have the same goals).

*Partner involvement in retirement planning*. Participants completed the same single-item measure of partner involvement in retirement planning as Study 1, rephrased for a retrospective assessment (i.e., 1 = my partner was not directly involved in planning at all, 7 = my partner was directly involved in planning a great deal).

*Actual ease of retirement*. A single-item measure assessed how easy participants felt their transition into retirement had been (“All things considered, how easy would you say your transition to retirement has been? When answering, please think about all aspects of your life since you have retired.”; 1 = extremely difficult, 7 = extremely easy).

*Current well-being*. A 29-item measure of current well-being [43] was included in Study 2 (*α* = .95; “I am physically healthy”, “Life has meaning for me”; 1 = strongly disagree, 6 = strongly agree). Items were averaged and higher scores reflected greater current subjective well-being.

*Self-esteem*. Participants completed the same 10-item measure of trait self-esteem as Study 1 (*α* = .90; e.g., “I feel that I am a person of worth, at least on an equal basis with others”, 1 = strongly disagree, 7 = strongly agree).

### Results

#### Descriptives

Consistent with Study 1, on average participants reported relatively high retrospective cognitive interdependence (*M =* 5.63, *SD =* 1.18), and they believed that their interdependence had become stronger post-retirement (*M =* 5.73, *SD =* 1.11) and that their partners were highly instrumental to their goals post-retirement (*M* = 2.58, *SD* = 1.91). Likewise, participants on average reported that their partners had been directly involved in retirement planning pre-retirement (*M* = 5.55, *SD* = 1.74) and that their post-retirement goals (i.e., current) were aligned with their partner’s (*M* = 5.61, *SD* = 1.39). Finally, participants reported that their transition into retirement had been relatively easy (*M* = 5.28, *SD* = 1.42), and that their well-being was generally good (*M =* 4.81, *SD =* .75). See Table 3 for the descriptive statistics and correlations for the measures in Study 2.

**Table 3:** Study 2 correlations and descriptive statistics.

Measure	1	2	3	4	5	6	7	8	9	10	11
1. Cognitive Interdependence	--										
2. Changes to Interdependence	.40***	--									
3. Post-Retirement Goal Instrumentality	.36***	.39***	--								
4. Goal Alignment	.55***	.44***	.68***	--							
5. Partner Involvement	.42***	.25*	.53***	.56***	--						
6. Ease of Retirement	.11	.02	.37***	.32**	.17	--					
7. Well-Being	.26*	.14	.39***	.36***	.10	.51***	--				
8. Self-Esteem	.29**	.05	.13	.36*	-.04	.36***	.59***	--			
9. Gender	.13	.08	.14	.18†	.14	.16	.15	.17	--		
10. Years to Retirement	.05	.02	-.05	-.26*	-.12	-.17	-.15	-.11	-.11	--	
11. Age	.13	.07	-.12	-.12	-.13	-.13	.05	.07	-.11	.41***	--
*M*	5.63	5.73	2.58	5.61	5.55	5.28	4.81	5.91	--	4.46	67.95
*SD*	1.18	1.11	1.91	1.38	1.74	1.42	.75	1.03	--	4..64	3.92
*Observed Range*	2.25–7	3.00–7	-4.29–5	1–7	1–7	1–7	2.17–6	2.1–7	--	0–2	62–78

Note.

†p<0.1

*p < .05

**p < .01

*** p < .001. Table 3 presents the bivariate correlations between measures, as well as their mean (M), standard deviation (SD), and observed range in the sample.

#### Primary analyses

Linear regression analyses were again used to examine associations between retrospective cognitive interdependence pre-retirement and perceived changes to cognitive interdependence following retirement, post-retirement goal instrumentality, goal alignment post-retirement, having involved the partner in planning decisions, ease of the transition into retirement and subjective well-being. Gender, years since retirement, and self-esteem were included as covariates (see Table 4 Model 1 for model coefficients). R (v. 4.0.5) was used for all analyses in this study.

**Table 4:** Model coefficients: Study 2.

	Predictors				Covariates					
	Cognitive Interdependence		Post-Retirement Goal Alignment		Self-Esteem		Gender		Years since Retirement	
Dependent Variables	*b*	*t*	*b*	*t*	*b*	*t*	*b*	*t*	*b*	*t*
**Model 1**										
Changes in Interdependence Post-Retirement	.39	3.90***			-.08	-0.7	.10	.43	.00	0.01
Partner Involvement in Planning	.69	4.65***			-.34	-2.01*	.32	.94	-.06	-1.58
Post-Retirement Goal Instrumentality	.57	3.29**			.02	.08	.32	.78	-.02	-.56
Post-Retirement Goal Alignment	.62	5.88***			.09	.78	.19	.78	-.08	-3.04**
Ease of Retirement	.02	.13			.44	3.03**	.22	.75	-.04	-1.23
Subjective Well-being	.07	1.13			.40	5.94***	.04	.26	-.02	-1.03
**Model 2**										
Ease of Retirement	-.17	-1.15	.30	2.32*	.42	2.90**	.16	.56	-.02	-.46
Subjective Well-being	-.01	-.12	.12	2.02*	.39	5.86***	.01	.10	-.005	-.36

Note.

†p<0.1

*p < .05

**p < .01

*** p < .001. Table 4, Model 1 presents the regression model coefficients for retrospective cognitive interdependence pre-retirement predicting changes in cognitive interdependence, actual partner involvement in retirement planning, post-retirement goal instrumentality, actual post-retirement goal alignment, and actual ease of retirement, and subjective well-being, controlling for trait self-esteem, gender and years since retirement as covariates. Model 2 presents the exploratory analyses for actual goal alignment in retirement predicting actual ease of retirement, and subjective well-being, controlling for trait self-esteem, gender and years since retirement as covariates.

Consistent with the findings from our sample of people approaching retirement in Study 1, among those who had recently retired, greater cognitive interdependence pre-retirement was associated with having more directly involved their partner in retirement planning decisions, greater goal instrumentality and goal alignment post-retirement, and having become even more interdependent following the transition into retirement (*ps <* .001; see Table 4 Model 1). Although a traditional alpha criterion of *p* < .05 is used to interpret the findings, these hypothesis tests also pass the Bonferroni corrected alpha criterion threshold for significance of *p* < .008 for this study. However, inconsistent with the hypotheses, retrospective cognitive interdependence pre-retirement was not associated with the actual ease of the transition to retirement, nor with subjective well-being.

#### Exploratory analyses

Given the associations between cognitive interdependence and goal alignment in Studies 1 and 2, and prior research pointing to associations between goal alignment and goal outcomes and well-being (e.g., [39, 40]), we explored whether post-retirement goal alignment might explain the perceived ease of retirement and well-being post-retirement. Exploratory regression analyses suggest that actual post-retirement goal alignment was indeed significantly associated with the ease of retirement and subjective well-being (*ps <* .05; see Table 4 Model 2). Thus, although high cognitive interdependence is important in setting expectations and intentions for the transition into retirement, having post-retirement goals that are aligned may be more pragmatically associated with how people actually experience retirement. However, it is important to reiterate that these additional tests were post-hoc exploratory tests, and therefore caution should be used in their interpretation. A traditional alpha criterion of *p* < .05 is used to interpret the findings, however, these hypothesis tests do not pass the Bonferroni corrected alpha criterion threshold for significance of *p* < .008 for this study. Thus, caution should be used in interpreting these findings.

## Study 3

The findings from Studies 1 and 2 suggest that people who are more cognitively interdependent in their relationship are more likely to directly involve their partner’s in their retirement planning decisions, see their partner as more instrumental to their goals pre- and post-retirement and feel as though their post-retirement goals are more aligned. However, while cognitive interdependnece was associated with anticipated ease of retirement among soon-to-be retirees, exploratory analyses suggest that it was post-retirement goal alignment, which reflected perceived current goal alignment for retirees, that was associated with how easy the transition into retirement had been, as well as current subjective well-being.

Cognitively interdependent people have positive expectations and intentions for retirement, but it is the extent to which they can coordinate their goals leading into retirement which appear to matter for actual retirement outcomes. One possible explanation for the discrepancy is that cognitive interdependence helps people more communally navigate the inevitable interdependence dilemmas (e.g., when partners disagree about what retirement will be like) and worries (e.g., ambivalence about retirement) that arise when people plan for the future. When people feel that their partners are not facilitating their goals—such as when their goals for retirement clash—they may respond by distancing themselves from their partners and relationships [44]. However, people can also be drawn to seek comfort and safety in their relationships during times of uncertainty [45]—such as when people feel conflicted about the transition into retirement. People who see their partners as pluralistically enmeshed in their lives and identities (i.e., high cognitive interdependnece) are motivated to signal commitment to the relationship [46], anticipate similarity with and positivity from their partners [47], and prioritize communal behaviors and select situations that actually increase interdependnece between partners [20] even when they are feeling vulnerable [48]. During the transition into retirement, this can be accomplished by involving partners more directly in retirement planning behavior (a communal response). These responses should help strengthen interdependence in the relationship, as well as increase future goal alignment as people select situations that involve their partner and coordinate with their partner in their planing for the future.

The goal of Study 3 was to test whether people relatively high in cognitive interdependence responded to retirement-based uncertainty (i.e., goal discordance; ambivalence about retirement), by increasing their partner’s involvement in their retirement planning decisions. Study 3 also tested whether people relatively high in cognitive interdependence fluidly compensate for retirement-based uncertainty by anticipating greater goal alignment in the future. We expected retirement ambivalence, reminders of retirement goal discordance and cognitive interdependence would interact, such that feeling generally uncertain about retirement would amplify the threat of a partner’s goals clashing with one’s own, and that those relatively high (compared to low) in cognitive interdependence would cope with these retirement-based uncertainties through the communal-response of involving their partners in their retirement planning to a greater extent and anticipating greater goal alignment in the future.

### Methods

#### Participants

In order to obtain a similar cohort of participants as Study 1, 364 adults based in the United States between the ages of 50 and 70, who were currently in a romantic relationship, and who had not yet retired were recruited via Amazon MTURK to take part in this study online. One hundred and two participants were excluded for reporting they were not in a relationship (n = 3), for being younger than 50 (n = 3) or older than 70 (n = 9), for not identifying as a man or woman (n = 2), for not completing the survey through to the end (n = 31), and for not following instructions during the goal priming task (did not understand task, n = 16; reported no goal alignment/misalignment, n = 10; did not mention partner, n = 17; provided single-word responses or phrases unrelated to the prompt (e.g., “very good”, “nice”), n = 11), leaving a final sample of 262 participants. Participants were 59.29 years old on average (*SD* = 4.92), 82% identified as white, and 63% were women. The majority (73%) were married (2% casually dating, 22% in an exclusive/committed dating relationship, 3% engaged) and had been in their relationships an average of 22.71 years (*SD* = 14.22).

#### Materials & procedures

This research received ethical approval from the University of Essex Ethics Subcommittee 3. Participants gave written consent to take part in the study; they were provided with an information sheet and consent form on the first page of the survey, before the screening questions. Participants who did not select “I consent” from the list on the consent form immediately had their session terminated and were thanked for their time and interest. All eligible participants completed background demographics questions, followed by the measures of self-esteem (*α* = .92) and cognitive interdependence (*α* = .82) from Study 1. Next, participants were randomly assigned to a retirement goal prime condition. In the goal concordance condition, participants were asked to write about an instance in which their goals and their partner’s goals for retirement were aligned. In the goal discordance condition, participants were asked to write about an instance in which their goals and their partner’s goals for retirement were not aligned. Following the goal prime, all participants completed new measures of retirement ambivalence, the likelihood of involving partner in planning decisions, their support for egalitarian decision-making, and how prepared they were for retirement, as well as the same measures of direct partner involvement, anticipated post-retirement goal alignment, and anticipated ease of retirement from Study 1.

*Retirement ambivalence*. Participants completed a 6-item measure asking them to reflect on how much ambivalence they feel about their retirement (*α* = .91; adapted from [49]; “When I think about my retirement, I feel. . . uncomfortable”, 1 = not at all, 7 = very). Responses were averaged across items and higher scores reflect greater ambivalence.

*Likelihood of involving partner in retirement planning activities*. Participants completed a 33-item measure assessing the likelihood that they would involve their partner in different retirement planning decisions (*α* = .96; adapted from [50]; “Participate in a workshop, seminar, or course on retirement”, 1 = not at all likely to involve my partner, 7 = extremely likely to involve my partner). Responses were averaged across items and higher scores reflect greater likelihood of involving the partner in retirement planning activities.

*Prepared for retirement*. Participants completed a single-item measures that assessed how prepared they are for retirement (1 = extremely unprepared, 7 = extremely prepared).

*Egalitarian decision-making*. Participants completed a single-item measure in which they were asked whether they agreed more with the statement that it is better for partners to have equal say in retirement planning decisions or for one partner to take the lead (1 = completely agree it is better for one partner to take the lead, 7 = completely agree it is better for decisions to be made equally).

### Results

#### Descriptives

On average, participants reported that they were highly interdependent (*M = 5*.*70*, *SD = 0*.*92*) and that they believed their post-retirement goals were relatively aligned with their partner’s (*M* = 5.23, *SD* = 1.58). They indicated that they were somewhat likely include their partners in a variety of retirement planning activities (*M* = 4.74, *SD* = 1.17), and preferred an egalitarian approach to decision-making (*M* = 6.09, *SD* = 1.40). Likewise, participants on average reported that they had largely directly involved their partners in their retirement plans thus far (*M* = 5.29, *SD* = 1.70). Finally, participants felt somewhat prepared for (*M* = 4.64, *SD* = 1.56), and somewhat ambivalent about their retirements (*M* = 3.66, *SD* = 1.57), and anticipated somewhat easy transitions into retirement (*M* = 4.66, *SD* = 1.57). Table 5 presents the descriptive statistics for the study measures.

**Table 5:** Study 3 correlations and descriptive statistics.

Measure	1	2	3	4	5	6	7	8	9	10	11	12
1. Cognitive Interdependence	--											
2. Prepared for Retirement	.09	--										
3. Anticipated Ease of Retirement	.08	.56***	--									
4. Retirement Ambivalence	-.07	-.52***	-.61***	--								
5. Likelihood of Involving Partner in Planning	.29***	.20**	.12†	-.19**	--							
6. Egalitarian Decision-Making	.04	.05	.09	.03	.08	--						
7. Direct Partner Involvement	.38***	.31***	.14*	-.20**	.50***	.28***	--					
8. Anticipated Post-Retirement Goal Alignment	.34***	.25***	.19**	-.28***	.44***	.18**	.66***	--				
9. Self-Esteem	.29***	.29***	.18**	-.32***	.24***	.06	.17**	.18**	--			
10. Gender	-.02	-.18**	-.10	.14*	-.07	.21**	.02	-.06	.000	--		
11. Age	.08	.09	.04	-.02	.000	.09	.08	.02	.07	.09	--	
12. Years to Retirement	.002	-.33***	-.25***	.25***	.01	-.07	-.10	-.08	-.09	-.08	-.53***	--
13. Condition	.04	-.10	-.08	.14*	-.02	-.08	-.03	-.24***	-.06	-.06	-.02	.03
*M*	5.68	4.63	4.64	3.66	4.74	6.07	5.27	5.20	5.74	--	59.17	7.11
*SD*	.92	1.56	1.58	1.57	1.17	1.42	1.71	1.58	1.07	--	4.95	5.25
*Observed Range*	1.50–7	1–7	1–7	1–7	1.45–7	1–7	1–7	1–7	2.30–7	--	50–70	0–25

Note.

†p<0.1

*p < .05

**p < .01

*** p < .001. Table 5 presents the bivariate correlations between measures, as well as their mean (M), standard deviation (SD), and observed range in the sample.

### Primary analyses

Linear regression analyses were used to predict likelihood of partner involvement, direct involvement of the partner so far, perceived goal alignment post-retirement, perceived preparedness for retirement, and anticipated ease of retirement from (1) the main effects of goal prime condition (-1 = goal concordance; 1 = goal discordance), cognitive interdependence (mean centered), and retirement ambivalence (mean centered); (2) all possible 2-way interactions; and, (3) all possible 3-way interactions, controlling for gender, years to retirement, and self-esteem as covariates. These control variables are consistent with those used in Studies 1–2, and self-esteem has been previously shown to moderate how people respond to relationship threats and negative information about a partner (for example: [51, 52]. Table 6 presents the model coefficients for the main effects and interaction models. R (v. 4.0.5) was used for all analyses in this study.

**Table 6:** Model coefficients: Study 3.

	Dependent Variables											
	Likelihood of Involving Partner in Retirement Planning Activities		Direct Partner Involvement in Retirement Planning		Egalitarian Decision-Making		Anticipated Post-Retirement Goal Alignment		Prepared for Retirement		Anticipated Ease of Retirement	
Regression Models	*b*	*t*	*b*	*t*	*b*	*t*	*b*	*t*	*b*	*t*	*b*	*t*
Main Effects Model												
Cognitive Interdependence	.32	4.13***	.64	5.86***	.07	.72	.57	5.80***	.01	.11	.05	.61
Goal Prime Condition	-.01	-.14	-.04	-.36	-.09	-1.04	-.38	-4.33***	-.08	-.99	-.01	-.11
Retirement Ambivalence	-.10	-2.07*	-.15	-2.24*	.04	.62	-.20	-3.16**	-.38	-6.68***	-.58	-10.49***
Self-Esteem	.15	2.08*	.03	.34	.08	.94	-.02	-.24	.17	2.11*	-.06	-.73
Gender	.06	.77	-.05	-.50	-.29	-3.20**	.08	.93	.25	3.00**	.05	.66
Years to Retirement	.01	.84	-.02	-.93	-.02	-.96	-.01	-.60	-.07	-4.22***	-.03	-2.07*
**Interaction Model **												
Cognitive Interdependence	.31	4.02***	.62	5.54***	.07	.73	.57	5.79***	.02	.19	.04	.47
Condition	.01	.15	-.003	-.03	-.07	-.84	-.35	-4.05***	-.07	-.90	.01	.08
Ambivalence	-.12	-2.40*	-.19	-2.76**	.02	.39	-.23	-3.60***	-.39	-6.75***	-.59	-10.47***
Self-Esteem	.17	2.37*	.07	.65	.08	.95	.01	.08	.17	2.06*	-.02	-.28
Gender	.04	.58	-.07	-.68	-.29	-3.28**	.07	.72	.25	2.99**	.04	.44
Years to Retirement	.01	1.00	-.01	-.74	-.01	-.71	-.01	-.57	-.07	-4.36***	-.03	-1.81†
Interdependence x Condition	.01	.14	-.05	-.48	-.17	-1.82†	.05	.50	-.02	-.26	.16	.32
Interdependence x Ambivalence	-.02	-.32	.06	.78	-.05	-.85	-.02	-.36	-.01	-.25	.02	.32
Condition x Ambivalence	-.02	-.40	-.07	-1.12	.02	.29	-.11	-1.93†	-.13	-2.51*	.057	1.09
Interdependence x Condition x Ambivalence	.12	2.27*	.19	2.64**	.11	1.71†	.15	2.37*	.03	.50	.09	1.60

Note.

†p<0.1

*p < .05

**p < .01

*** p < .001. Table 6 presents the regression model coefficients for the main effects of cognitive interdependence, condition, and ambivalence (main effects model), and their two- and three-way interactions (interaction model), predicting likelihood of involving partner in planning activities, having directly involved the partner in planning activities, egalitarian decision-making, anticipated post-retirement goal alignment, feelings of preparedness for retirement, and anticipated ease of retirement, controlling for gender, years to retirement and self-esteem as covariates.

#### Main effects model

Consistent with Studies 1 and 2, higher cognitive interdependence was associated with a higher perceived likelihood of participants involving their partner in future retirement planning behaviors, higher ratings of having directly involved their partners in retirement planning decisions, and higher anticipated goal alignment post-retirement (*ps <* .001). Unlike in the previous studies, cognitive interdependence was not associated with ease of retirement, nor was it associated with the new measures of perceived retirement preparedness or perceptions regarding power divisions in planning decisions. The main effect of goal discordance condition was only significant for anticipated goal alignment, suggesting that the manipulation (which asked participants to describe a goal concordant or goal discordant event) made people feel as though their partner’s retirement goals were less aligned with their own when they were asked to write about retirement goal discordance. Finally, the main effect of retirement ambivalence was significantly and negatively associated with perceived likelihood of involving the partners and direct partner involvement suggesting that feeling ambivalent about retirement may lead people to withdraw from their partners rather than engage with them when it comes to planning for the future (*ps <* .05). Retirement ambivalence was also significantly and negatively associated with anticipated goal alignment, preparedness, and anticipated ease of retirement, suggesting that ambivalence about retirement is associated with less positive perceptions of their futures (*ps <* .001). There was no association with power divisions in planning decisions. Although a traditional alpha criterion of *p* < .05 is used to interpret the findings, not all of the tests for the main effects pass the Bonferroni corrected alpha criterion threshold for significance of *p* < .008 for this study. Notably, the main effect of ambivalence predicting likelihood of involving the partner and direct partner involvement should be interpreted with caution.

#### Interaction model

**Fig 1:**
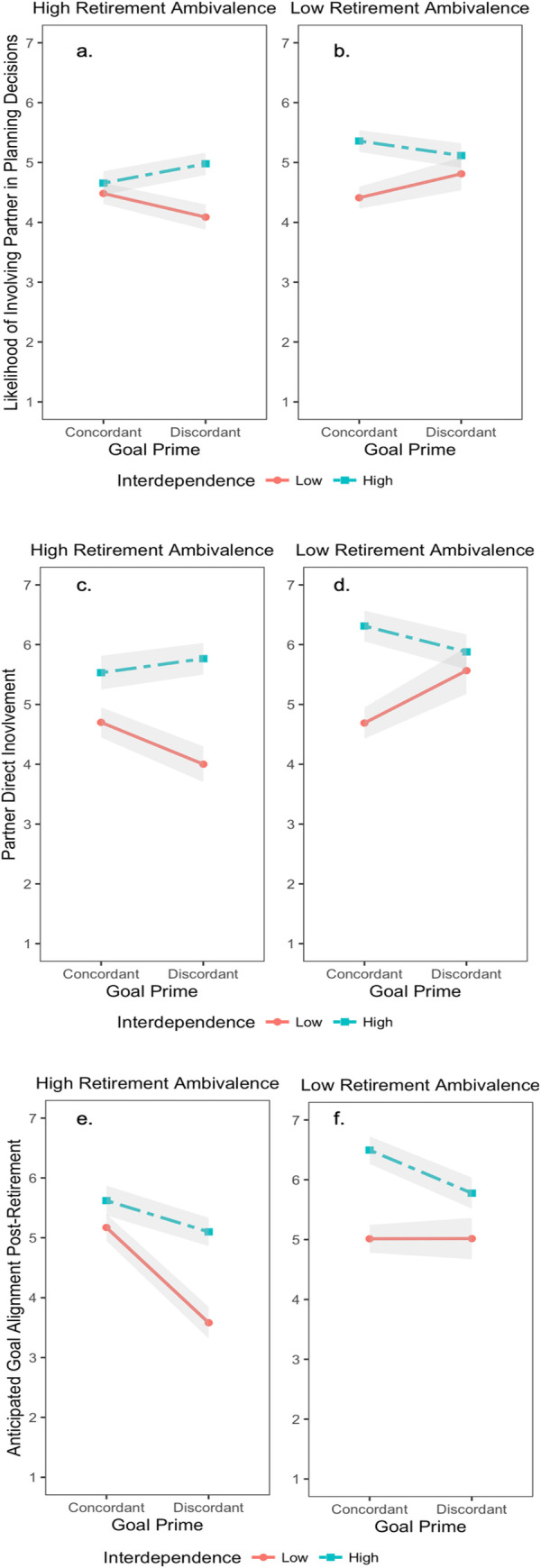
The cognitive interdependence by condition by ambivalence interaction across outcomes. Fig 1A and 1B present the interaction predicting the likelihood of involving partner in planning decisions; Fig 1C and 1D the interaction predicting direct partner involvement in retirement planning; and, Fig 1E and 1F the interaction predicting anticipated post-retirement goal alignment with partner. High and low retirement ambivalence and cognitive interdependence are plotted at ±1SD from the mean.

Next, we tested our hypothesis that cognitive interdependence may help people cope with feelings of uncertainty about retirement (i.e., goal conflict; ambivalence) by testing the 3-way cognitive interdependence by condition by ambivalence interaction. This significantly predicted likelihood of involving the partner in retirement planning decisions, direct partner involvement, and anticipated post-retirement goal alignment (*ps <* .05; Fig 1). Although a traditional alpha criterion of p < .05 is used to interpret the findings, the interaction effects do not meet the Bonferroni corrected alpha criterion threshold for significance of *p* < .008 for this study and should therefore be interpreted with caution.

*Simple effects of cognitive interdependence*. To test for a potential buffering effect of cognitive interdependence, we decomposed the positive interactions to test for the simple effects [53] for those high (+1*SD*) and low (-1*SD*) in retirement ambivalence, and those in the concordant and discordant goal conditions. First, when retirement ambivalence was high and goal discordance had been primed, significant simple effects of cognitive interdependence emerged predicting likelihood of involving the partner in planning behaviors (*b =* .48, *t*(250) = 3.10, *p =* .002), direct partner involvement in planning decisions (*b =* .96, *t*(249) = 4.32, *p <* .001), and anticipated post-retirement goal alignment (*b =* .82, *t*(249) = 34.19, *p <* .001). Thus, when people felt more uncertain about their retirements (primed with goal discordance and highly ambivalent), those high in cognitive interdependence responded by incorporating their partners in their planning decisions to a greater extent and anticipating more goal concordance in the future compared to those who were relatively lower in cognitive interdependence. When ambivalence was high, but goal concordance had been primed, the simple effects of cognitive interdependence no longer significantly predicted likelihood of involvement (*b =* .09, *t*(250) = .74, *p =* .46) and anticipated goal alignment (*b =* .24, *t*(249) = 1.51, *p =* .13). However, the simple effect of cognitive interdependence did significantly predict direct partner involvement for those high in ambivalence in the goal concordance condition (*b =* .45, *t*(249) = 2.48, *p =* .01). Thus, despite their latent uncertainty, the reminder of the goal concordance meant that people with high cognitive interdependence no longer fluidly compensated by drawing the partner into their planning decisions in the future or anticipating even greater goal alignment than those relatively lower in cognitive interdependence. However, they continued to believe that they had already directly involved their partner in their planning decisions to a greater extent.

When ambivalence was low, the cognitive interdependence by condition interactions were not significant for predicting likelihood of involvement and anticipated goal alignment (*ps>*.15), but were significant for direct partner involvement (*b = -*.36, *t*(249) = -2.10, *p =* .04). When ambivalence was low and goal discordance had been primed, the simple effect of cognitive interdependence predicting direct partner involvement was not significant (*b =* .17, *t*(249) = .61, *p =* .54), but was significant when goal concordance had been primed (*b =* .88, *t*(249) = 4.04, *p <* .001). Thus, in general, in the absence of latent retirement uncertainty goal discordance did not motivate people with relatively high cognitive interdependence to fluidly compensate by drawing closer to their partner, even following a reminder of a retirement goal conflict, nor did it make them retrospectively believe they had directly involved their partners to a greater/lesser extent compared to those relatively lower in cognitive interdependence. However, when uncertainty about retirement was low overall (low ambivalence, goal concordance), those relatively high in cognitive interdependence believed they had directly involved their partners to a greater extent than those relatively low in cognitive interdependence. This suggests that goal conflicts during the planning for retirement may lead those high in cognitive interdependence to feel as though they have not involved their partners to the extent that they would like to believe.

It must be noted that the 2-way cognitive dissonance by condition interaction for those high in ambivalence was significant for goal alignment (*b* = .29, *t*(249) = 2.26, *p* = .02), but marginal for likelihood of involvement (*b* = .19, *t*(250) = 1.92, *p* = .056) and direct partner involvement (*b* = .25, *t(*249) = 1.75, *p* = .08) so the simple slopes should be interpreted with caution.

## General discussion

The transition into retirement can feel like a momentous, yet daunting, life event for many adults [9, 10]. Fortunately, for those in romantic partnerships, it is one that they will not have to navigate alone. Cognitive interdependence motivates people to view their relationships and their partners as highly interconnected with their sense of self, effectively blurring the lines between the “self” and the “partner” [4, 7]. This collectively-oriented self-concept leads people to believe that their partner shares the same vision of the world around them as they do [5]. Past research suggests that believing that the relationship operates as a single goal-pursuing unit, as well as having partners who are aligned with and facilitate personal goals, is associated with greater goal progress, in addition to happier and more stable relationships [6, 8, 40, 54]. We proposed that in a retirement context, greater cognitive interdependence would motivate people to believe partners have shared goals and futures, eliciting behaviors (e.g., direct planning) that facilitate shared outcomes (i.e., actual goal alignment and goal instrumentality in retirement).

The findings from our two correlational studies—one with people approaching retirement and one with retirees—and one experimental study, support our hypothesis that cognitive interdependence can be an important interpersonal characteristic that helps people navigate the transition into retirement with their romantic partners. First, among soon-to-be retirees, greater cognitive interdependence was associated with more positive expectations for retirement, perceiving their partners to be more instrumental and aligned with their personal goals, and more active involvement of their partners in retirement planning decisions. Among recent retirees, cognitive interdependence was still associated with perceiving partners as more instrumental and aligned with personal goals, and more involved in past planning decisions. However, it was the extent to which post-retirement goals were seen as aligned which was associated with actual ease of the transition into retirement and post-retirement subjective well-being.

This discrepancy between interpersonal factors that contribute to outcomes pre-transition and then weaken post-transition is consistent with research on other major life events that suggest working models of partners and relationships can shift throughout transitional periods [55]. Our experimental study with soon-to-be retirees helps shed some preliminary light on why this discrepancy between Study 1 and Study 2 may emerge. Not everyone experiences the transition into retirement the same way. Some may experience more interdependence dilemmas or uncertainty about their future. However, our findings suggest that people with greater cognitive interdependence may respond more communally to these challenges by drawing their partners into their retirement planning decisions to a greater extent, and anticipate the realignment of their goals in the future. Thus, cognitive interdependence may be a resource that motivates more communal and collectivistic intentions towards to retirement planning. Although not directly tested in the current studies, if these intentions become overt behaviors [56], then responding to ambivalence by involving the partner directly in planning decisions to a greater extent should help align goals and expectations for life post-retirement especially in partnerships where people have more collectivistic mindsets [57].

Overall, our studies are consistent with past work pointing to the benefits of being cognitively interdependent with a partner, and extend previous findings to the retirement context. Past research suggests that greater interdependence motivates people to behave more communally in their relationships, prioritizing prosocial behaviors that enhance the relationship rather than just the self [25–28]. This has previously been linked to risk-reducing decisions and coping strategies in health consequences, with positive outcomes for both the individual and the relationship [29, 30]. Our findings further suggest that cognitive interdependence likely leads to similarly communally-focused behaviors and decision-making before and after retirement. However, our findings are also consistent with past work showing that people may feel prepared and well-aligned with their partner’s vision for retirement, when in actuality their expectations differ substantially [38]. Rather, our findings suggest that while cognitive interdependence can help people assume the best possible outcome and engage in behaviors that facilitate those outcomes (e.g., involving the partner in planning activities), what really matters is having a partner who is aligned with one’s goals post-retirement [8, 39, 40].

Sociodemographic influences associated with graying societies has prompted a recent increase in government and corporate policies focused on helping people adequately plan for this next phase of their lives. However, most of these policies focus on financial preparedness alone [15]. As a result, these policies ignore the reality that retirement transitions are not solitary adventures. Most people will transition into retirement alongside their romantic partners, who may or may not have the same goals and expectations for retired life. Consequently, the road to retirement should be more successful for those who have partners who facilitate and are directly involved in the retirement planning process, than those whose partners thwart their retirement goals and planning. Instead of focusing exclusively on financial preparedness, policies should also consider ways in which they can help couples become more instrumental to one another’s goals so that they may reap the benefits of heading towards retirement in the same direction with an instrumental ally who shares their vision.

### Sample & generalizability

All of our studies recruited samples from Amazon MTURK Workers. Consistent with previous research (e.g., [41]), participants came from diverse sociodemographic backgrounds and employment sectors, including skilled and unskilled workers, with and without postsecondary education, and from lower-, middle-, and higher-income backgrounds. Furthermore, the sample sizes in all three studies were sufficient to detect the medium to large correlations (88% power to detect *rs*> = .33). Although our studies were adequately powered and were socio-demographically diverse, a larger sample would be needed to examine more nuanced effects, such as whether sociodemographic factors moderate the associations between cognitive interdependence, retirement planning decisions, and ease of retirement (see for example: [58]). The studies would also benefit from being replicated in other cultures where retirement experiences may differ from the North American context (e.g., [59]). Nonetheless, the current studies provide important insights into how relationship partners can shape preparation, experiences, and well-being during the transition to retirement.

### Limitations & future directions

It is important to note the limitations of the current studies. First, Studies 1 and 2 relied on single-item measures of goal alignment, partner involvement in retirement planning, and expected/experienced ease of the transition into retirement, though Study 3 used a 33-item measure of retirement planning activities. Although single-item measures are sometimes discouraged because their internal reliability cannot be estimated, they also offer some advantages. First, they lessen respondent fatigue by reducing questionnaire length and complacency due the feeling among participants that they have already answered the same question [60]. Second, single-item measures are appropriate when researchers are interested in more global—rather than domain specific or multi-faceted—constructs, such as a person’s expectations for the future [61, 62]. Nonetheless, future studies would benefit from examining the reliability of these measures over time and including multifaceted measures that may tap into more nuanced or domain-specific aspects of these constructs.

Furthermore, the current set of studies are limited in their scope to draw conclusive claims because they rely on cross-sectional analyses of people before and after they have transitioned into retirement. Although this provides important insights into which factors are essential and both stages (e.g., cognitive interdependence; perceived goal alignment), longitudinal research is needed to track how couples navigate the transition into retirement, and how their evolving goals interact throughout this important life event. Study 2 also relied on retrospective assessments of cognitive interdependence, and how involved partners had been in the planning process. Retrospective assessments can be biased by the desire to remember the past as rosier than it was. These limitations would also be addressed by a longitudinal study which would provide the opportunity to measure goal instrumentality at pre-retirement, and its influence on post-retirement outcomes for the same individuals over time. However, the combined findings of the current studies suggest that cognitive interdependence is an important relationship characteristic influencing how people engage with their partners regarding retirement planning, and their expectations and experiences with retirement. Finally, this research only provided a snapshot into people’s well-being post-retirement. A longitudinal study would enable researchers to identify which processes are most important at different stages throughout the transition into retirement, and whether factors that matter within the initial six to 12 months are as important several years later. Furthermore, these studies relied on only one partner’s perspective. Though perceptions can be biased, they are also strongly linked with eliciting desired behaviors in partners [51, 63], and actor perceptions can be an even better predictor of relationship outcomes than partner effects [64]. However, studies that collect dyadic data where *both* partners’ perceptions, goals, and engagement in retirement planning decisions are assessed would provide additional insight into how cognitive interdependence contributes to a successful transition to retirement.

Future research should also seek to better understand how cognitive interdependence changes within relationships throughout transitional periods such as retirement, and what the consequences of these changes may be. Retirement not only disrupts former routines, but also people’s sense of self [13]. Since cognitive interdependence reflects the extent to which people see the self-and-relationship as a pluralistic collective [7], substantial changes to the self or challenges during the transition may affect how integrated the partner is in the self-concept post-retirement. Holding evaluatively different attitudes about a partner pre- and post-transition is associated with defensive and self-protective prioritization [55], which could undermine communal behaviors and contribute to additional stress during retirement. The extent to which poor retirement planning and goal misalignment contributes to these discordant partner attitudes pre- and post-transition should be studied further.

Finally, there needs to be a better understanding of the implications of this work for people who are no longer in a romantic relationship relative to those who are [65]. Although people in romantic relationships have been shown to benefit across a variety of health and well-being metrics later in life when compared to people who are single [66, 67], these benefits depend on relationship quality [68, 69]. Thus, additional research is needed to determine whether retirement outcomes are better for single people than those who have relatively poor goal alignment or are less cognitively interdependent in their relationships, or whether less interdependence and a less aligned partner is better than no partner at all when it comes to navigating the stresses and challenges retirement presents.

## Conclusions

As couples walk hand-in-hand into the golden years, it is important that they are walking in the same direction. The findings from this research suggests that cognitive interdependence—a mental representation of a collective self-in-relationship—is important for the transition into retirement. Prior to retirement, people who were more cognitively interdependent in their relationships believed that their goals for retirement were more aligned with their partners and were more likely to directly involve their partners in retirement planning, particularly when facing retirement-centered challenges. They also believed their transition into retirement would be relatively easier. In retirement, being cognitively interdependent with the partner was still associated with positive outcomes, but it was the extent to which couples had aligned their post-retirement goals which contributed to successful retirement transitions and well-being. Thus, finding ways to behave collectively and align goals pre-retirement may pay dividends in the next phase of life couples navigate together.
